# Two-sample Mendelian Randomization to evaluate the causal relationship between inflammatory arthritis and female-specific cancers

**DOI:** 10.1186/s12967-024-05765-9

**Published:** 2024-10-24

**Authors:** Christa Meisinger, Simone Fischer, Tracy O’Mara, Dennis Freuer

**Affiliations:** 1 Epidemiology, Medical Faculty, University of Augsburg, University Hospital of Augsburg, Stenglinstr. 2, 86156 Augsburg, Germany; 2 Genetics and Computational Biology Department, QIMR Berghofer Medical Research Institute, Brisbane, 4006 Australia

**Keywords:** Mendelian randomization, Ovarian cancer, Breast cancer, Autoimmune disease, Ankylosing spondylitis, Psoriatic arthritis, Rheumatoid arthritis

## Abstract

**Background:**

There is evidence that inflammatory arthritis in the form of ankylosing spondylitis (AS), psoriatic arthritis (PsA), and rheumatoid arthritis are both positively and negatively associated with certain female-specific cancers. However, the study results are very heterogeneous.

**Methods:**

Based on up to 375,814 European women, we performed an iterative two-sample Mendelian randomization to assess causal effects of the occurrence of the inflammatory arthritis on the risk of female-specific cancer in form of breast, endometrial, and ovarian cancer sites as well as their subtypes. Evidence was strengthened by using similar exposures for plausibility or by replication with a subsequent meta-analysis. P-values were Bonferroni adjusted.

**Results:**

Genetic liability to AS was associated with ovarian cancer (OR = 1.03; 95% CI: [1.01; 1.04]; $\:{P}_{adj}$=0.029) and liability to PsA with breast cancer (OR = 1.02; CI: [1.01; 1.04]; $\:{P}_{adj}$=0.002). Subgroup analyses revealed that the high-grade serous ovarian cancer (OR = 1.04; CI: [1.02; 1.06]; $\:{P}_{adj}$=0.015) and the ER- breast cancer (OR = 1.04; CI: [1.01; 1.07]; $\:{P}_{adj}$=0.118) appeared to drive the observed associations, respectively. No further associations were found between the remaining inflammatory arthritis phenotypes and female-specific cancers.

**Conclusions:**

This study suggests that AS is a risk factor for ovarian cancer, while PsA is linked to an increased breast cancer risk. These results are important for physicians caring women with inflammatory arthritis to advise their patients on cancer screening and preventive measures.

**Supplementary Information:**

The online version contains supplementary material available at 10.1186/s12967-024-05765-9.

## Background

The prevalence of autoimmune diseases is estimated to be about 10%, and it is predicted to continue to increase worldwide [1] with consequently enormous health and economic burdens [2, 3]. Their prevalence can vary widely in different regions and countries, depending on ethnicity, age, and other demographic factors [1, 4]. It is also known that women are more often affected by autoimmune diseases than men. However, the reasons for this are not yet clear [5].

There is evidence that autoimmune diseases are associated with an increased risk of cancer, which may be due to an underlying dysregulation of the immune system or the therapies used to treat the diseases [6–9]. Common representatives of autoimmune diseases are inflammatory joint diseases, whose connection with the occurrence of various cancers has been the subject of several previous studies [10–12]. However, the risk for female cancers in these patients has been understudied. Previous observational investigations on this topic have focused primarily on rheumatoid arthritis (RA), one of the most common autoimmune diseases, and female cancers; most studies suggested reduced or no risk of endometrial [13, 14], breast [14, 15], cervical [15], and ovarian cancer [14]. So far, there is scarce data on whether women with other forms of inflammatory arthritis such as psoriatic arthritis (PsA) and ankylosing spondylitis (AS) are more or less likely to develop breast, endometrium, and ovarian cancers [16, 17].

Despite previous findings, it is difficult to establish a causal relationship between inflammatory arthritis and the occurrence of various cancers in women because of the possibility of unmeasured confounding factors or reverse causality in observational studies.

We therefore performed a two-sample Mendelian randomization (MR) study to investigate the causal effects of different types of inflammatory arthritis, namely RA, AS, and PsA on breast, ovarian, and endometrial cancer and their subgroups.

## Methods

### Study design

MR is an instrumental variable analysis method in which genetic variants are used as instruments for an exposure to infer causality with an outcome of interest. The allocation of the genetic variants is assumed to be naturally randomized according to Mendel’s laws and thus not influenced by any confounding factor of the exposure-outcome association. Because of this randomization process, this study design is less prone to unobserved confounding or reverse causality compared to the observational study design. The following three core assumptions should ensure the definition of an instrumental variable for each of the genetic variants in a MR: Genetic instruments (1) have to be associated with the risk factor of interest (relevance assumption), (2) must not be associated with confounding factors of the exposure-outcome association (independence assumption), and (3) must influence the outcome only via exposure and not via any other path (exclusion restriction assumption).

### Study samples

All analyses were based on female individuals of European ancestry. To minimize sample-overlap and in this way ensure an unbiased two-sample setting, we restricted the exposure datasets to non-UK Biobank participants, whereas outcome datasets did not contain participants from the FinnGen study.

### Exposures

The summary-level data regarding the RA phenotype were taken from two different sources considering participants of European ancestry. The GWAS of Okada et al. included 29,880 cases and 73,758 controls [18] and the FinnGen study included 12,555 cases and 240,862 controls [19] [Table 1]. The FinnGen database was also used to extract data for both AS and AS under a strict definition (as a subgroup of AS), containing 2,860 and 1,193 cases, respectively. In this way we were able to compare the strengths and consistencies of results. We had a similar rationale in case of psoriasis phenotypes where PsA, the autoimmune disease of interest, was a subset of psoriasis. With the exception of PsA, which we took from the 8th round of the FinnGen study, the remaining phenotypes were derived from the 9th round [Table 1].

**Table 1:** Description of exposure-datasets and associated independent genetic instruments based on the genome-wide association threshold of $\:P=5\cdot\:{10}^{-8}$

Phenotype	Source	Cases	Controls	Associated SNPs
Ankylosing spondylitis	FinnGen	2,860	270,964	14
Ankylosing spondylitis (strict def.)	FinnGen	1,193	374,621	11
Psoriasis	FinnGen	9,267	364,071	33
Psoriatic arthritis	FinnGen	2,912	330,975	14
Rheumatoid arthritis	Okada et al.	29,880	73,758	46
Rheumatoid arthritis	FinnGen	12,555	240,862	27

Abbreviations: SNPs Single Nucleotide Polymorphisms

### Outcomes

Three women-specific cancers and their subgroups were considered as outcomes [Table 2]. The GWAS for breast cancer and its subtypes, estrogen receptor-positive (ER+) and -negative (ER-) comprised 122,977, 69,501, and 21,468 cases, respectively, each compared with 105,974 controls [20]. The endometrial cancer GWAS conducted by O’Mara et al. included 12,906 cases and 108,979 controls [21]. The endometrioid endometrial carcinoma (EEC), which typically has a good prognosis, and the non-endometrioid endometrial carcinoma (NEEC), with a worse prognosis [22], were used in the subgroup analyses. In contrast to the main analysis, the subgroup analyses did not include UK Biobank individuals. The ovarian cancer GWAS including 25,509 cases and 40,941 controls and its subgroups endometrioid ovarian cancer, clear cell ovarian cancer, and low- and high-grade serous ovarian carcinomas conducted by Phelan et al. [23] were accessed through the IEU OpenGWAS database [24, 25].

**Table 2:** Description of outcome-datasets used in main and subgroup mendelian randomization analyses

Cancer site	Subgroup	Source	Cases	Controls
Breast		Michailidou et al.	122,977	105,974
	ER+		69,501	105,974
	ER-		21,468	105,974
Endometrial		O’Mara et al.	12,906	108,979
	EEC		8,758	46,126
	NEEC		1,230	35,447
Ovary		Phelan et al.	25,509	40,941
	Low-grade serous		1,012	40,941
	High-grade serous		13,037	40,941
	Endometrioid		2,810	40,941
	Clear cell		1,366	40,941

Abbreviations: ER+, estrogen receptor-positive; ER-, estrogen receptor-negative; EEC, endometrioid endometrial carcinoma; NEEC, non-endometrioid endometrial carcinoma

### Instrument selection

Due to the relevance assumption, single nucleotide polymorphisms (SNPs) that were associated with the respective exposure were selected based on the genome-wide association threshold of $\:P=5\cdot\:{10}^{-8}$. Independency of genetic instruments was ensured by performing PLINK clumping to prune SNPs in linkage disequilibrium (LD) within a 10,000 kb window using a conservative clumping cutoff of $\:{r}^{2}$=0.001. During the harmonization process, palindromic SNPs with a minor allele frequency $\:>$ 0.42 were removed from the analyses, and a proxy-search was performed for instruments not found in the respective outcome datasets, restricting the LD to $\:{r}^{2}>0.8$.

### Statistical analyses

The inverse variance-weighted (IVW) method with modified second-order weights within the radial MR framework was used as the principal approach embedded into an iterative setting. In this way, we were able to focus on outliers contributing to global heterogeneity in each iteration with respect to their individual Cochran’s $\:Q$ statistics (based on an $\:{\alpha\:}_{Q}$=0.01) and the number of instruments. Results from the first and last iterations were compared to check the distortion as well as consistency and therefore the robustness of estimates.

To assess different heterogeneity patterns and rule out biases related to horizontal pleiotropy, we performed a range of pleiotropy-robust methods. Under the InSIDE assumption, the MR-Egger provides a consistent estimate even when the intercept (added as an additional parameter to a random effects IVW model) is different from zero (i.e. directional pleiotropy). The weighted median and mode approaches also produce consistent estimates when less of more than 50% of instruments are invalid, respectively. The MR-RAPS (Robust Adjusted Profile Score) approach was performed to account for weak instruments bias and extreme outliers in case of balanced pleiotropy. Finally, in each iteration the MR-PRESSO was used to identify outliers and, if necessary, to test for distortion of estimates before and after outlier removal in each iteration.

Presence of substantial and directional (i.e. unbalanced) pleiotropy was investigated applying the MR-PRESSO global test and the MR-Egger intercept test, respectively. Additionally, Cochran’s $\:Q$ and Ruecker’s $\:{Q}^{{\prime\:}}$ statistics as well as their differences and ratios were calculated and tested.

To strengthen the evidence, we pooled the results from both RA-datasets using a fixed-effect meta-analysis. With regard to the evidence in AS patients, we compared the results of two datasets. The first dataset included all cases within the FinnGen cohort, while the second dataset was restricted to cases with a stricter definition. We then interpreted only those associations with consistent point estimates.

Presented P-values were Bonferroni adjusted to correct for multiple testing for 44 null hypotheses due to four exposure sets and eleven outcomes (three main outcomes and seven subgroup analyses). Estimates were presented as ORs, 95% CIs and Bonferroni-adjusted P-values.

All analyses were performed using the open-source statistical software R (version 4.2.2). For the most part, the following software packages (version number) were used: LDlinkR (1.3.0), mr.raps (0.2), MendelianRandomization (0.9.0), MRPRESSO (1.0), RadialMR (1.1), MVMR (0.4), TwoSampleMR (0.5.6), data.Table (1.14.8), dplyr (1.1.2), and ggplot2 (3.4.3).

## Results

After the clumping procedure, between 11 and 46 associated independent SNPs were available to be considered as potential genetic instruments for the corresponding exposures at the beginning of the iterative analyses [Table 1]. The model-specific number of genetic variants before and after outlier removal in up to three iterations were listed in Supplementary Table 1. In the following, the estimates on the OR scale represent the average change in the outcome per 2.72-fold increase in the prevalence of the exposures from the final models after outlier removal [Supplementary Table 2].

### Main analyses

**Fig. 1:**
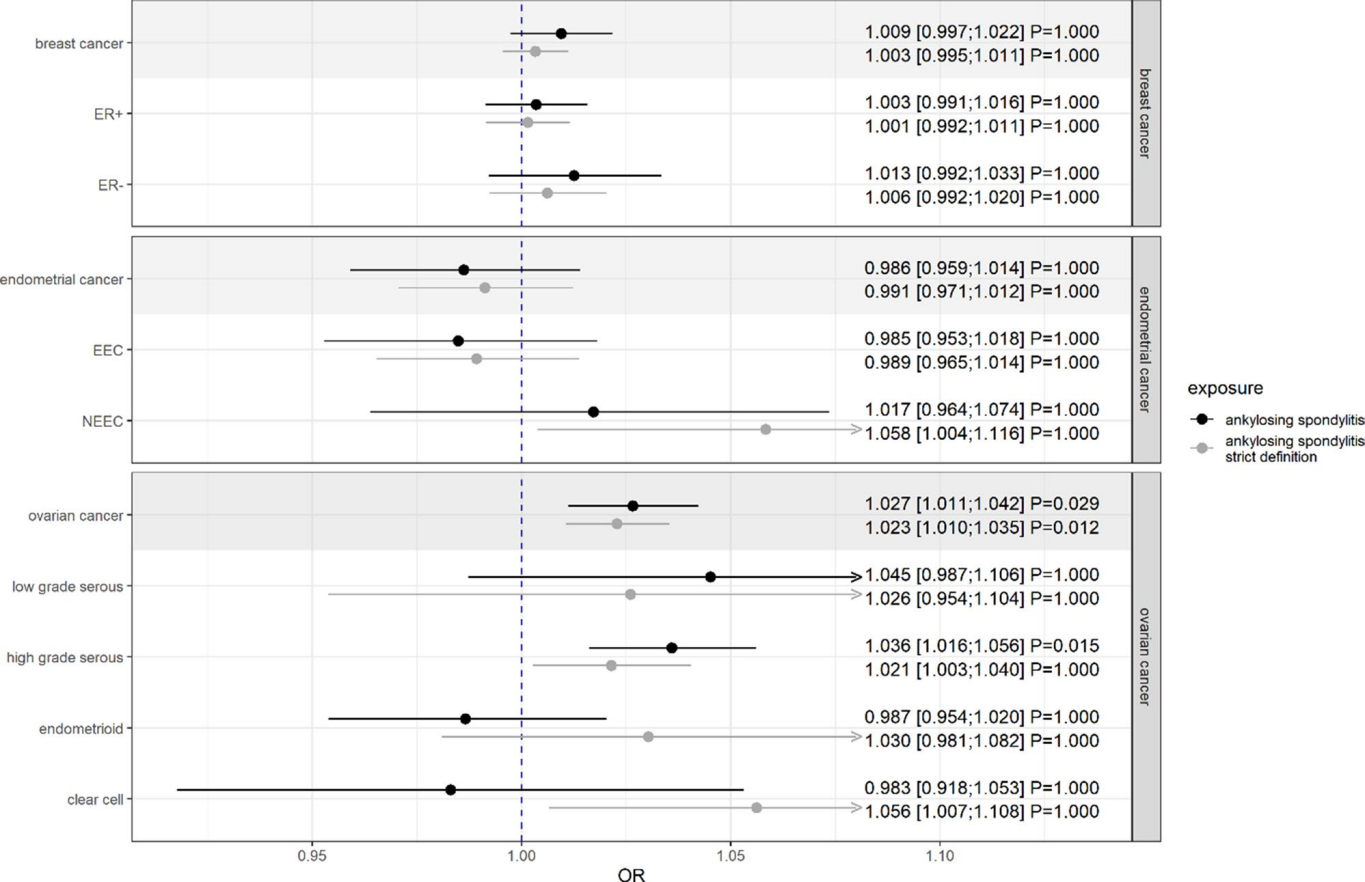
Estimates for the causal effects of ankylosing spondylitis (using the usual and a strict definition) on female-specific cancers given as odds ratios (ORs) and 95% confidence intervals. Presented P-values are Bonferroni-adjusted. A gray background represents the main and the unshaded background the subgroup analyses of the respective cancer site

Genetic liability to AS was positively associated with the occurrence of ovarian cancer (OR = 1.03; CI: [1.01; 1.04]; $\:{P}_{adj}$=0.029) [Fig. 1]. The result held also for the strict definition of AS (OR = 1.02; CI: [1.01; 1.04]; $\:{P}_{adj}$=0.012). Subtype analyses revealed a positive association with high-grade serous ovarian cancer (OR = 1.04; CI: [1.02; 1.06]; $\:{P}_{adj}$=0.015) with a consistent estimate in case of strict definition (OR = 1.02; CI: [1.00; 1.04]; $\:{P}_{adj}\approx\:$1). Contradictory estimates were observed in endometrioid and clear cell ovarian cancers with a negative point estimate for AS and a positive for the strict definition of AS. Regarding this fact and the adjusted P-values of approximately 1, no clear conclusion can be drawn for the both subtypes.

**Fig. 2:**
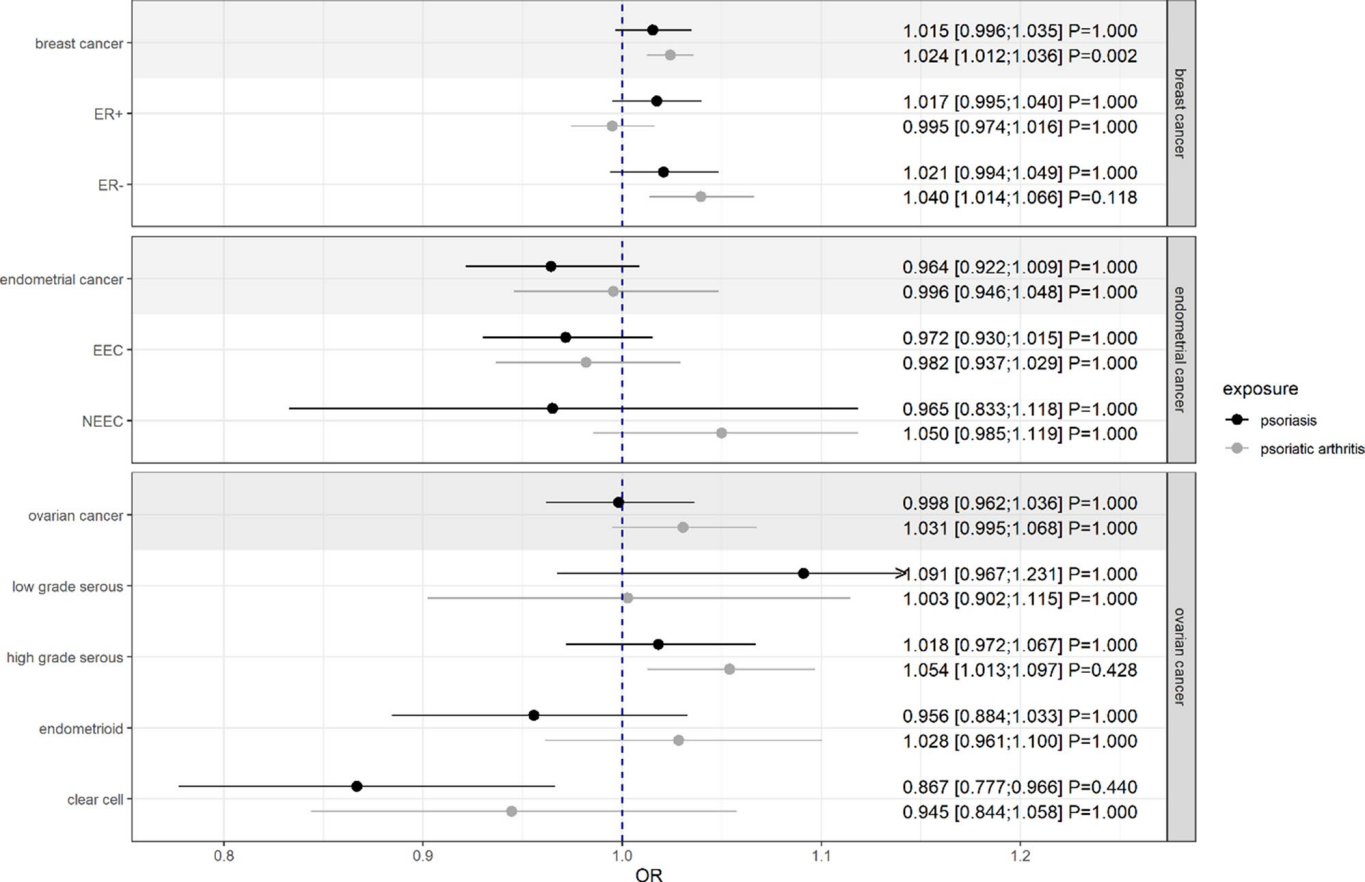
Estimates for the causal effects of psoriasis and psoriatic arthritis on female-specific cancers given as odds ratios (ORs) and 95% confidence intervals. Presented P-values are Bonferroni-adjusted. A gray background represents the main and the unshaded background the subgroup analyses of the respective cancer site

While there were no notable associations between genetic liability to psoriasis and major cancer risk, genetically liability to PsA was found to be associated with breast cancer (OR = 1.02; CI: [1.01; 1.04]; $\:{P}_{adj}$=0.002) that appeared to be driven by its relationship to the ER- subtype (OR = 1.04; CI: [1.01; 1.07]; $\:{P}_{adj}$=0.118) [Fig. 2]. Although there was little evidence of an association with ovarian cancer (OR = 1.03; CI: [1.00; 1.07]; $\:{P}_{adj}\approx\:$1), a suggestive positive association of PsA with high-grade serous subtype and a negative association of psoriasis with the clear cell subtype was observed, indicating a potential heterogenous influence on cancer subtypes. Again, inconsistent estimates were observed for the endometrioid ovarian cancer.

**Fig. 3:**
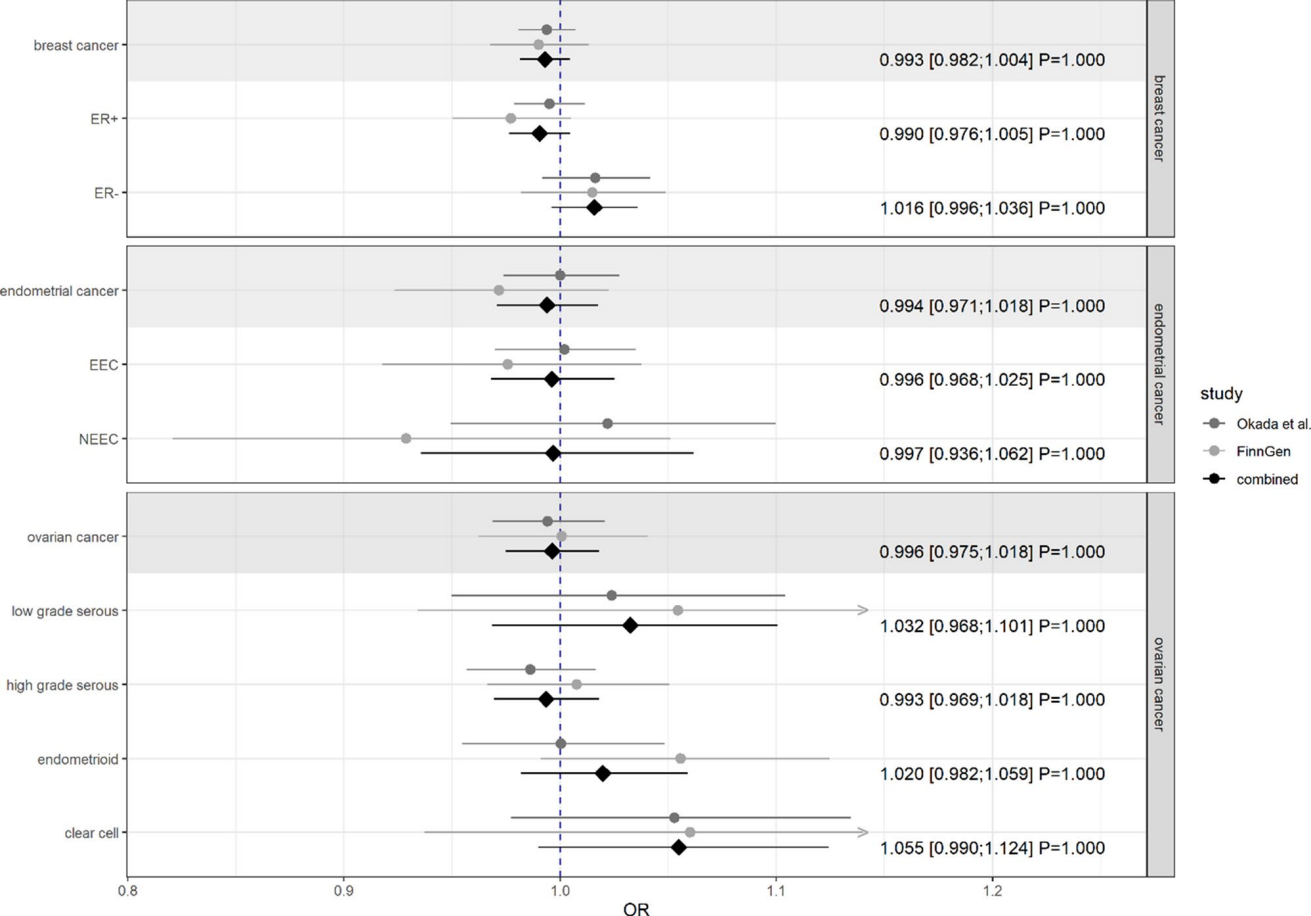
Estimates for the causal effects of rheumatoid arthritis on female-specific cancers, consisting of two individual studies and a pooled estimate from a fixed-effect meta-analysis. Results are presented as odds ratios (ORs), 95% confidence intervals, and Bonferroni-adjusted P-values. A gray background represents the main and the unshaded background the subgroup analyses of the respective cancer site

No evidence of an inverse association between genetically liability to RA and female-specific cancer risk was found in either the individual studies or the meta-analysis that included both studies (i.e. Okada et al. and FinnGen) [Fig. 3].

### Robustness and heterogeneity

Looking at the iterative approach, the point estimates before outlier removal agreed with the final results without a substantial distortion effect [Supplementary Fig. 1]. Also, the pleiotropy-robust approaches used in sensitivity analyses fully supported the results from main analyses in terms of consistency and magnitude of point estimates [Supplementary Figs. 2–4]. Notably, except MR-Egger, which is known to have a low statistical power, all pleiotropy-robust approaches were statistically significant for the findings mentioned above based on an $\:\alpha\:$-level of 0.05.

Regarding the notable associations, there was no indication for substantial heterogeneity in the final models on an $\:\alpha\:$-level of 0.05 [Supplementary Table 3]. However, heterogeneity (especially directional pleiotropy) was observed particularly in RA-breast cancer models, indicating biased estimates. In these cases, the weighted median and weighted mode approaches suggested a negative association between RA and breast cancer [Supplementary Fig. 4].

In summary, we found a positive genetic association between AS and ovarian cancer, possibly due to the high-grade serous subtype. Furthermore, PsA appeared to be a risk factor for the development of breast cancer, particularly the ER- subtype. Uncertainties existed in the inverse relationship between RA and breast cancer.

## Discussion

The present study using a two-sample mendelian randomization approach found a causal relationship between PsA and an increased risk of breast cancer, as well as between AS and the risk of ovarian cancer. There was no evidence for further causal relationships between PsA, psoriasis, AS, and RA with endometrial, breast, and ovarian cancer.

A systematic review and updated meta-analysis investigating the impact of RA on the risk of breast cancer, found no overall increased cancer risk in RA patients. However, in sub-group analysis, breast cancer risk was increased in non-Caucasians patients while it was decreased in Caucasians [10]. Recently, a large observational study from UK reported that women with RA have a lower risk of endometrial cancer and a modestly decreased risk of breast cancer. No association with ovary cancer was found in that study [26]. Contrary, a Mendelian randomization study in European and East Asian populations found a suggestive relationship of genetic liability to RA and ovarian cancer and no association with endometrial and breast cancer in a meta-analysis combining different GWAS data from European populations [27]. The results of our study confirm these prior findings regarding the association between RA and endometrium and breast cancer. We found no link between RA and ovarian cancer in European women.

To date, there are only few studies on the association between PsA and female cancers [9]. In a Canadian cohort including 680 patients with PsA, the frequency of different cancers in comparison to the general population was determined [16]. In that study, the cancer incidence did not differ from that seen in the general population. Contrary, another population-based cohort study from the US reported a 41% increased incidence of breast cancer in comparison to the general population [28]. Our result due to PsA and breast cancer is in accordance with the finding from the latter study. Additionally, our study revealed that this relationship was mainly due to the association with ER- breast cancer [29]. As far as we aware, there are no previous studies on the association between PsA and ovarian and endometrium cancer, so our results on this question are the first and no comparisons can be made with other studies. Furthermore, we did not find an association between psoriasis and any of the considered female-specific cancer sites, a result that is consistent with previous findings from observational studies [17].

Studies of whether patients with AS are at increased risk for cancer are rare. A retrospective cohort study from Western Australia reported no increased risk of cancer diagnosis in AS patients [30]. These findings are also supported by a population-based Swedish cohort study that found no increased overall cancer risk in patients with AS [31]. A study by Hemminki et al. including close to 200,000 patients from Sweden with any diagnosis of 33 autoimmune diseases observed no increased risk for breast and ovarian cancer in patients with AS, but a decreased standardized incidence ratio for endometrium cancer [17]. In the present study, a causal relationship between AS and ovarian cancer, in particular the high-grade serous subtype, the most common form of epithelial ovarian cancers, was found.

### Evidence for cancer subtypes

In previous studies investigating the relationship between autoimmune diseases and female-specific cancers, the analyses were generally not stratified by subtype. This is probably due to the small number of cases of the individual subtypes. So far there are no studies available which investigated whether rarer endometrial cancer subtypes, particularly non-endometrioid endometrial cancers (NEEC), which account for less than 20% of endometrial cancer cases and include more aggressive subtypes such as serous papillary carcinoma, clear cell carcinoma and carcinosarcoma would be more likely associated with inflammatory arthritides [32]. The same applies to the relationship with breast cancer subtypes. Breast cancer is a heterogeneous disease with multiple molecular/clinical subtypes based on factors such as hormone receptor status and human epidermal growth factor receptor 2 (HER2) protein expression [33]. Approximately 80% of breast cancers are breast tumors with hormone receptors on the cell surface and are associated with the best prognosis of all subtypes. The extent to which inflammatory arthritides are associated with the subtypes HER2-positive breast cancer and triple-negative (basal-like) breast cancer, which are generally associated with a poorer prognosis and which exhibit different degrees of inflammation [34, 35], cannot currently be answered due to a lack of studies. This also applies to the association of inflammatory arthritis and subtypes of endometrial cancer, which have genomic features that overlap with the very aggressive, estrogen-negative, basal-like subtypes of breast cancer [36].

### Biological mechanisms

Factors that may contribute to an increased or decreased cancer risk in patients with inflammatory arthritis include, for example, immune dysregulation, hormonal exposures, genetic factors and medication [37–40]. In addition, a complex interplay between genetic and environmental factors could be involved in the development of malignancies in patients with autoimmune diseases [41]. Furthermore, epigenetic changes may influence the risk of cancers in these patients [42].

There is evidence that a dysregulation of immune cells in patients with autoimmune diseases may be a trigger or risk for the development of malignancies [40, 43, 44]. Prior research has shown that the tumor necrosis factor-alpha (TNF-α) pathway, and interleukin-23 (IL-23), and interleukin-17 (IL-17) axis play an important role in inflammatory arthritis [45]. In addition, the molecular mechanism of HLA-B27 misfolding appears to be associated with immune activation [46]. Also, a large number of toll-like receptors (TLR) and other innate receptors are found in inflamed joints [47]. While the innate immune cells are able to respond, they also maintain an inflammatory immune memory, which underlines the role of non-immune and innate immune cells in autoinflammatory diseases. Inflammation is suggested to contribute to the development and progression of various female malignancies [48]. IL-17 has been identified in various tumors, including ovarian cancers [49, 50]. Several studies suggested potent anti-tumor functions of IL-17 and IL-17-producing cells [51–53], whereas other reports indicated that IL-17 promotes tumor growth [54, 55]. Thus, at present, the exact mechanisms underlying the dysregulation of the immune system in certain inflammatory arthritides that play a role in carcinogenesis and tumor development in females are still controversial.

Hormonal factors could also play a role regarding the association between genetic liability to inflammatory arthritis and female cancers. Both ovarian and breast cancer are influenced by various hormonal signaling pathways [39, 56, 57] and there is evidence that estrogens may play an important role in ovarian and breast carcinogenesis [57, 58]. Estrogens are also involved in the pathogenesis of inflammatory arthritis [59]. Due to the present findings, women with PsA have an increased risk of breast cancer, in particular, the ER-negative subtype. So far, less is known on the role of sex hormones in patients with PsA [59] and there is no possible explanation for this result at this time.

### Strengths and limitations

One of the major strengths of this work is the MR study design, which have several advantages over conventional observational studies and is less likely to yield biased estimates due to unmeasured confounding or residual bias, inherent in most of the studies reviewed. We extended this study design through the use of an iterative approach and performed bias assessment by focusing on outliers responsible for horizontal pleiotropy. Together with a series of pleiotropy-robust methods and several tests, our approach ensured robust and reliable estimates. We also used more than one data set per exposure where possible and focused on the plausibility of our results. The use of the largest GWAS available at the time of this study increased the statistical power.

However, in the present study, it was not possible to consider treatment effects in inflammatory arthritis. Treatment of chronic inflammatory diseases such as RA, AS, and PsA is nowadays frequently performed with tumor necrosis factor-α inhibitors (TNFi). Patients treated with TNFi have not been found to have an increased risk of cancer overall or for the six most common cancers (including breast cancer) compared with TNFi-naive patients with any of the chronic inflammatory diseases mentioned above [60]. Furthermore, although MR estimates based on binary exposures provide a valid test for a causal relationship, they are generally imprecise [61]. Therefore, the results from this study should not be interpreted on the basis of their magnitude, but only as a test for a causal effect. Finally, we only included European women, so it is not possible to generalize the results to other ethnic groups, which may be very different. The same applies to other cancer subgroups that could not be investigated in this study.

## Conclusions

The present study suggests that AS is a risk factor for ovarian cancer, while PsA is linked to an increased breast cancer risk. Future research in this area will help to unravel specific mechanisms and elucidate which factors are responsible for the associations between inflammatory arthritides and certain female-specific cancer entities. These risk factors are important for physicians caring women with inflammatory arthritis to understand in order to counsel these patients regarding cancer screening and preventive measures.

## Electronic supplementary material

Below is the link to the electronic supplementary material.

Supplementary Material 1

Supplementary Material 2

## Data Availability

Most of the analyses based on summary statistics from genome-wide association studies publicly available at the following resources. Summary statistics on rheumatoid arthritis can be accessed through the National Heart, Lung, and Blood Institute (https://grasp.nhlbi.nih.gov/FullResults.aspx). All datasets taken from FinnGen database can be found under the following link: https://www.finngen.fi/en/access_results. Summary-level data for breast cancer and ovarian cancer were taken from the Breast Cancer Association Consortium (https://bcac.ccge.medschl.cam.ac.uk/bcacdata/oncoarray/oncoarray-and-combined-summary-result/gwas-summary-results-breast-cancer-risk-2017/) and IEU OpenGWAS database (https://gwas.mrcieu.ac.uk/), respectively. The endometrial cancer dataset is not publicly available but may be obtained on request from authors conducting the GWAS.
