# Supplementary material for: Two-sample Mendelian Randomization to evaluate the causal relationship between inflammatory arthritis and female-specific cancers
Source: J Transl Med. 2024 Oct 24;22:962. doi: 10.1186/s12967-024-05765-9 (PMC11515448; doi:10.1186/s12967-024-05765-9)
Supplement: Supplementary file 2 — Supplementary Material 2 [file 12967_2024_5765_MOESM2_ESM.pdf]

Supplementary Material

Two-sample Mendelian Randomization to evaluate the causal relationship between inflammatory arthritis and female-specific cancers

Christa Meisinger*, Simone Fischer, Tracy O'Mara, Dennis Freuer

***Correspondence:** christine.meisinger@med.uni-augsburg.de

Table of contents

Tables

Table 1 Number of instruments and iterations
Table 2 Genetic variants identified as outliers
Table 3 Heterogeneity statistics from Mendelian randomization analyses

Figures

Figure 1 Results from iterative MR analyses
Figure 2 Pleiotropy robust methods for the association between ankylosing spondylitis and female-specific cancers
Figure 3 Pleiotropy robust methods for the association between psoriasis phenotypes and female-specific cancers
Figure 4 Pleiotropy robust methods for the association between rheumatoid arthritis and female-specific cancers

Supplementary Table 1 Number of instruments and iterations used in the iteraive MR analyses

Exposure	Outcome	n_{SNP} first	n_{SNP} last	iterations
ra	endometr.eec	43	43	1
ra	endometr.neec	43	41	2
ra	endometr	43	43	1
ra	bc	45	43	2
ra	bc.er+	44	43	2
ra	bc.er-	45	45	1
ra.fg	endometr.eec	26	24	2
ra.fg	endometr.neec	25	24	2
ra.fg	endometr	26	24	2
ra.fg	bc	26	23	2
ra.fg	bc.er+	26	23	3
ra.fg	bc.er-	26	26	1
pso.fg	endometr.eec	27	25	2
pso.fg	endometr.neec	27	26	2
pso.fg	endometr	27	25	2
pso.fg	bc	29	28	2
pso.fg	bc.er+	29	28	2
pso.fg	bc.er-	29	29	1
as.fg	endometr.eec	12	12	1
as.fg	endometr.neec	12	12	1
as.fg	endometr	12	12	1
as.fg	bc	14	14	1
as.fg	bc.er+	14	14	1
as.fg	bc.er-	14	14	1
as.strict.fg	endometr.eec	10	10	1
as.strict.fg	endometr.neec	10	10	1
as.strict.fg	endometr	10	10	1
as.strict.fg	bc	11	11	1
as.strict.fg	bc.er+	11	11	1
as.strict.fg	bc.er-	11	11	1
ra	Clear cell ovarian cancer	45	45	1
ra	High grade serous ovarian cancer	45	45	1
ra	Low grade serous ovarian cancer	45	45	1
ra	Ovarian cancer	45	45	1
ra.fg	Clear cell ovarian cancer	26	25	2
ra.fg	High grade serous ovarian cancer	26	24	2
ra.fg	Low grade serous ovarian cancer	26	25	2
ra.fg	Ovarian cancer	26	25	2
pso.fg	Clear cell ovarian cancer	29	29	1
pso.fg	High grade serous ovarian cancer	29	28	2
pso.fg	Low grade serous ovarian cancer	29	28	2
pso.fg	Ovarian cancer	29	28	2
as.fg	Clear cell ovarian cancer	14	14	1
as.fg	High grade serous ovarian cancer	14	13	2
as.fg	Low grade serous ovarian cancer	14	14	1
as.fg	Ovarian cancer	14	13	2
as.strict.fg	Clear cell ovarian cancer	11	10	2

as.strict.fg	High grade serous ovarian cancer	11	11	1
as.strict.fg	Low grade serous ovarian cancer	11	11	1
as.strict.fg	Ovarian cancer	11	11	1
psa.fg	endometr.eec	12	11	2
psa.fg	endometr.neec	12	12	1
psa.fg	endometr	12	11	2
psa.fg	bc	13	11	3
psa.fg	bc.er+	13	13	1
psa.fg	bc.er-	13	11	2
psa.fg	Clear cell ovarian cancer	13	13	1
psa.fg	Ovarian cancer	13	12	2
psa.fg	High grade serous ovarian cancer	13	11	2
psa.fg	Low grade serous ovarian cancer	13	12	2

Supplementary Table 2 Genetic variants identified as outliers within the iterative Mendelian randomization analyses. The iteration steps of exclusion, Q-statistics and the P-values of the instruments classified as outliers are presented.

Exposure	Outcome	iteration	SNP	Q_statistic	p.value
ra	endometr.neec	1	rs73432769	8,21	4,16E-03
ra	bc	1	rs10985070	12,62	3,82E-04
ra	bc	1	rs2476601	28,19	1,10E-07
ra	er+	1	rs2476601	16,15	5,84E-05
ra.fg	endometr.eec	1	rs11513729	24,28	8,35E-07
ra.fg	endometr.eec	1	rs16903065	29,31	6,16E-08
ra.fg	endometr.neec	1	rs34536443	8,31	3,95E-03
ra.fg	endometr	1	rs11513729	25,09	5,48E-07
ra.fg	endometr	1	rs16903065	25,85	3,69E-07
ra.fg	bc	1	rs114335056	8,41	3,73E-03
ra.fg	bc	1	rs2476601	31,73	1,77E-08
ra.fg	bc	1	rs78248443	7,55	5,99E-03
ra.fg	bc	1	rs9332735	6,74	9,43E-03
ra.fg	er+	1	rs114335056	9,03	2,66E-03
ra.fg	er+	1	rs2476601	19,43	1,04E-05
ra.fg	er+	2	rs7731626	6,77	9,28E-03
pso.fg	endometr.eec	1	rs16903065	29,16	6,65E-08
pso.fg	endometr.eec	1	rs2021511	8,70	3,17E-03
pso.fg	endometr.neec	1	rs34536443	7,79	5,24E-03
pso.fg	endometr	1	rs16903065	25,94	3,52E-07
pso.fg	endometr	1	rs2021511	8,88	2,88E-03
pso.fg	bc	1	rs7542079	6,64	1,00E-02
pso.fg	er+	1	rs9346778	8,15	4,31E-03
ra.fg	Clear cell ovarian cancer	1	rs16903065	9,14	2,50E-03
ra.fg	High grade serous ovarian cancer	1	rs16903065	82,83	8,93E-20
ra.fg	Low grade serous ovarian cancer	1	rs16903065	15,33	9,03E-05
ra.fg	Ovarian cancer	1	rs16903065	58,80	1,75E-14
pso.fg	High grade serous ovarian cancer	1	rs16903065	80,76	2,55E-19
pso.fg	Low grade serous ovarian cancer	1	rs16903065	14,83	1,18E-04

pso.fg	Ovarian cancer	1	rs16903065	58,97	1,60E-14
as.fg	High grade serous ovarian cancer	1	rs1611166	13,88	1,95E-04
as.fg	Ovarian cancer	1	rs1611166	11,37	7,45E-04
as.strict.fg	Clear cell ovarian cancer	1	rs139558884	7,14	7,52E-03
psa.fg	endometr.eec	1	rs1607120	29,53	5,51E-08
psa.fg	endometr	1	rs1607120	23,72	1,12E-06
psa.fg	bc	1	rs11085727	6,69	9,69E-03
psa.fg	bc	1	rs2546890	13,93	1,89E-04
psa.fg	bc	2	rs11085727	7,95	4,81E-03
psa.fg	er-	1	rs115174302	7,73	5,43E-03
psa.fg	er-	1	rs2546890	16,13	5,93E-05
psa.fg	Ovarian cancer	1	rs1607120	50,67	1,09E-12
psa.fg	High grade serous ovarian cancer	1	rs115174302	7,16	7,44E-03
psa.fg	High grade serous ovarian cancer	1	rs1607120	66,68	3,20E-16
psa.fg	Low grade serous ovarian cancer	1	rs1607120	15,82	6,96E-05

Supplementary Table 3 Results of the MR-PRESSO global and MR-Egger-intercept tests for detecting horizontal and directional pleiotropy and between SNP-heterogeneity based on the radial regression framework in the Mendelian randomization setting

Exposure	Outcome	PRESSO RSSobs	P_{RSSobs}	Radial-Egger intercept	P_{interc}	Cochrans Q	df	P_Q	Rueckers Q'	$P_{Q'}$	Q-Q'	$P_{Q-Q'}$	$^{Q'}/_{Q}$
ra	endometr.eec	47,131	0,358	-0,448	0,115	45,438	42	0,331	42,684	0,442	2,75	0,1	0,94
ra	endometr.neec	39,229	0,593	0,636	0,015	37,137	40	0,6	31,779	0,82	5,36	0,02	0,86
ra	endometr	44,151	0,476	-0,367	0,187	42,955	42	0,43	41,111	0,51	1,84	0,17	0,96
ra	bc	61,573	0,053	0,452	0,17	58,089	42	0,05	55,317	0,082	2,77	0,1	0,95
ra	bc.er+	66,638	0,02	0,494	0,144	64,466	43	0,019	61,089	0,036	3,38	0,07	0,95
ra	bc.er-	72,575	0,008	-0,022	0,949	68,719	44	0,01	68,707	0,01	0,01	0,91	1
ra.fg	endometr.eec	36,944	0,09	-0,673	0,126	32,688	23	0,087	29,291	0,171	3,4	0,07	0,9
ra.fg	endometr.neec	24,899	0,477	-0,062	0,869	23,817	23	0,414	23,791	0,415	0,03	0,87	1
ra.fg	endometr	33,979	0,132	-0,326	0,458	31,224	23	0,117	30,451	0,137	0,77	0,38	0,98
ra.fg	bc	44,02	0,05	0,969	0,027	33,835	22	0,051	26,485	0,231	7,35	0,01	0,78
ra.fg	bc.er+	45,97	0,03	0,818	0,082	36,997	22	0,024	31,688	0,083	5,31	0,02	0,86
ra.fg	bc.er-	30,171	0,351	-0,094	0,805	26,854	25	0,363	26,778	0,367	0,08	0,78	1
pso.fg	endometr.eec	19,96	0,801	-0,411	0,235	17,546	24	0,824	16,488	0,87	1,06	0,3	0,94
pso.fg	endometr.neec	36,593	0,131	0,419	0,384	33,291	25	0,124	32,159	0,153	1,13	0,29	0,97
pso.fg	endometr	27,546	0,392	-0,088	0,837	25,661	24	0,371	25,626	0,372	0,04	0,85	1
pso.fg	bc	34,408	0,254	-0,373	0,351	31,072	27	0,268	29,922	0,318	1,15	0,28	0,96
pso.fg	bc.er+	31,997	0,34	0,011	0,977	29,557	27	0,334	29,558	0,334	-0	1	1
pso.fg	bc.er-	23,725	0,804	-0,291	0,343	19,822	28	0,871	19,121	0,894	0,7	0,4	0,97
as.fg	endometr.eec	22,064	0,106	1,196	0,077	17,593	11	0,092	12,564	0,323	5,03	0,03	0,71
as.fg	endometr.neec	8,91	0,777	0,503	0,288	7,888	11	0,723	7,006	0,799	0,88	0,35	0,89
as.fg	endometr	20,329	0,13	1,167	0,082	17,193	11	0,102	12,407	0,334	4,79	0,03	0,72
as.fg	bc	27,839	0,056	0,042	0,951	24,189	13	0,029	24,185	0,029	0	0,95	1
as.fg	bc.er+	18,081	0,289	0,507	0,355	17,284	13	0,187	16,045	0,247	1,24	0,27	0,93
as.fg	bc.er-	31,646	0,083	-0,773	0,21	21,111	13	0,071	18,352	0,145	2,76	0,1	0,87
as.strict.fg	endometr.eec	10,336	0,514	0,211	0,807	9,361	9	0,405	9,281	0,412	0,08	0,78	0,99
as.strict.fg	endometr.neec	9,451	0,58	0,486	0,533	7,893	9	0,545	7,511	0,584	0,38	0,54	0,95
as.strict.fg	endometr	10,928	0,468	0,558	0,518	9,661	9	0,379	9,108	0,427	0,55	0,46	0,94
as.strict.fg	bc	10,423	0,586	-0,014	0,984	9,37	10	0,497	9,37	0,497	0	0,98	1

as.strict.fg	bc.er+	11,668	0,478	0,257	0,736	10,622	10	0,388	10,481	0,399	0,14	0,71	0,99
as.strict.fg	bc.er-	12,037	0,494	-0,802	0,227	8,949	10	0,537	7,507	0,677	1,44	0,23	0,84
ra	Clear cell ovarian cancer	53,909	0,198	0,682	0,018	51,637	44	0,2	45,275	0,419	6,36	0,01	0,88
ra	High grade serous ovarian cancer	59,678	0,096	-0,235	0,445	55,189	44	0,12	54,447	0,134	0,74	0,39	0,99
ra	Low grade serous ovarian cancer	41,905	0,658	-0,023	0,927	37,258	44	0,754	37,249	0,754	0,01	0,93	1
ra	Ovarian cancer	62,581	0,068	-0,01	0,974	57,951	44	0,077	57,95	0,077	0	0,98	1
ra.fg	Clear cell ovarian cancer	32,965	0,197	0,358	0,368	30,751	24	0,161	29,669	0,196	1,08	0,3	0,97
ra.fg	High grade serous ovarian cancer	24,612	0,524	-0,051	0,885	21,423	23	0,555	21,401	0,557	0,02	0,88	1
ra.fg	Low grade serous ovarian cancer	23,59	0,614	-0,282	0,385	21,31	24	0,62	20,586	0,663	0,72	0,4	0,97
ra.fg	Ovarian cancer	30,879	0,275	-0,28	0,468	28,371	24	0,245	27,708	0,273	0,66	0,42	0,98
pso.fg	Clear cell ovarian cancer	27,212	0,612	-0,154	0,677	25,996	28	0,573	25,86	0,581	0,14	0,71	1
pso.fg	High grade serous ovarian cancer	32,297	0,333	0,549	0,161	30,345	27	0,299	28,072	0,407	2,27	0,13	0,93
pso.fg	Low grade serous ovarian cancer	23,625	0,753	0,56	0,09	21,089	27	0,782	18,842	0,876	2,25	0,13	0,89
pso.fg	Ovarian cancer	28,944	0,467	0,358	0,339	26,986	27	0,465	26,003	0,518	0,98	0,32	0,96
as.fg	Clear cell ovarian cancer	21,17	0,179	0,042	0,944	18,961	13	0,124	18,952	0,125	0,01	0,92	1
as.fg	High grade serous ovarian cancer	11,561	0,678	-0,657	0,098	8,71	12	0,727	6,677	0,878	2,03	0,15	0,77
as.fg	Low grade serous ovarian cancer	10,317	0,795	0,299	0,453	8,981	13	0,774	8,556	0,806	0,43	0,52	0,95
as.fg	Ovarian cancer	8,61	0,845	-0,183	0,643	7,549	12	0,819	7,386	0,831	0,16	0,69	0,98
as.strict.fg	Clear cell ovarian cancer	9,519	0,581	-0,046	0,95	7,546	9	0,58	7,539	0,581	0,01	0,94	1
as.strict.fg	High grade serous ovarian cancer	10,757	0,56	-0,822	0,193	8,08	10	0,621	6,569	0,765	1,51	0,22	0,81
as.strict.fg	Low grade serous ovarian cancer	16,332	0,223	-0,545	0,521	13,563	10	0,194	12,882	0,23	0,68	0,41	0,95
as.strict.fg	Ovarian cancer	7,193	0,839	-0,35	0,495	4,916	10	0,897	4,643	0,914	0,27	0,6	0,94
psa.fg	endometr.eec	12,223	0,464	0,218	0,751	10,324	10	0,413	10,187	0,424	0,14	0,71	0,99
psa.fg	endometr.neec	5,082	0,964	0,418	0,246	3,662	11	0,979	3,182	0,988	0,48	0,49	0,87
psa.fg	endometr	22,787	0,116	0,723	0,405	17,179	10	0,071	15,745	0,107	1,43	0,23	0,92
psa.fg	bc	5,366	0,936	0,091	0,833	4,902	10	0,898	4,882	0,899	0,02	0,89	1
psa.fg	bc.er+	19,222	0,232	-0,232	0,739	16,12	12	0,186	15,954	0,193	0,17	0,68	0,99
psa.fg	bc.er-	14,324	0,5	-0,851	0,075	7,281	10	0,699	4,92	0,896	2,36	0,12	0,68
psa.fg	Clear cell ovarian cancer	17,264	0,276	-0,16	0,812	15,476	12	0,216	15,407	0,22	0,07	0,79	1
psa.fg	Ovarian cancer	16,138	0,36	-0,558	0,372	12,363	11	0,337	11,276	0,42	1,09	0,3	0,91
psa.fg	High grade serous ovarian cancer	16,154	0,386	-0,736	0,212	9,735	10	0,464	7,985	0,63	1,75	0,19	0,82
psa.fg	Low grade serous ovarian cancer	9,254	0,754	0,011	0,983	8,196	11	0,696	8,195	0,696	0	0,99	1

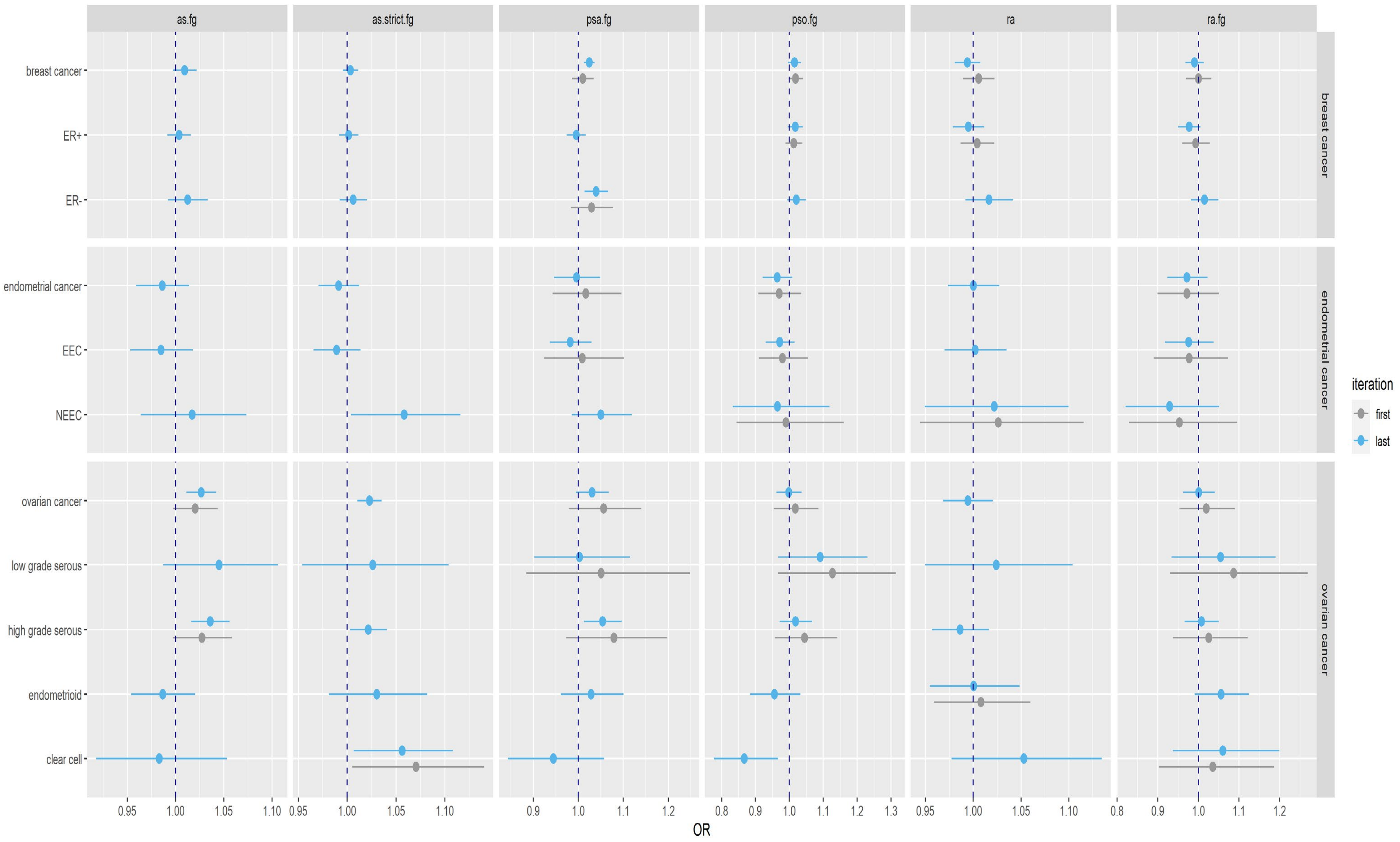

Supplementary Figure 1 Results of the first and last iterations of the iterative Mendelian randomization analyses investigating the impact of selected autoimmune diseases on female-specific cancer-risk. Causal estimates are given as odds ratios and 95 % confidence intervals.

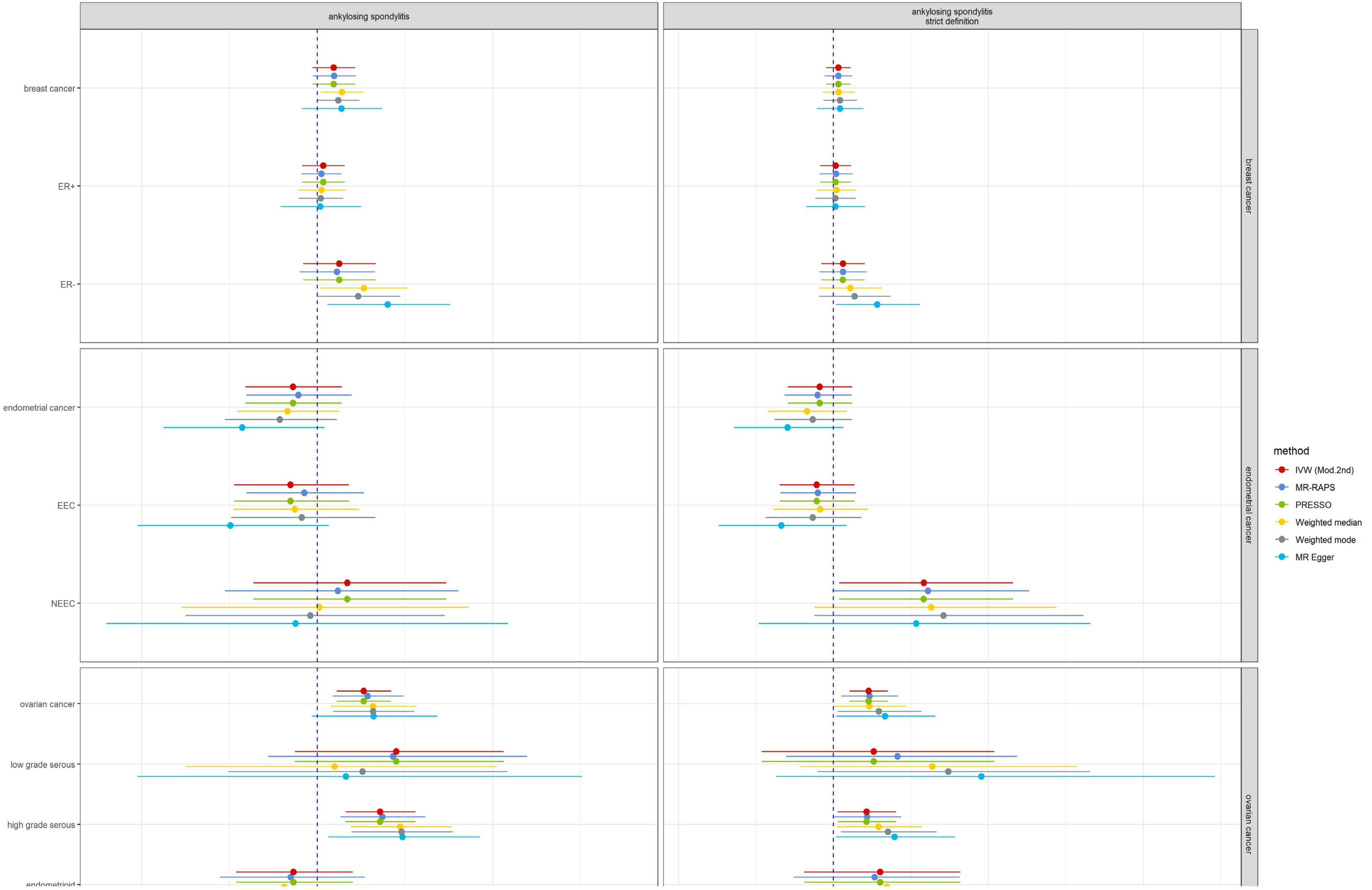
ankylosing spondylitis
ankylosing spondylitis
strict definition
breast cancer
ER+
ER-
endometrial cancer
EEC
NEEC
ovarian cancer
low grade serous
high grade serous
endometrioid
breast cancer
endometrial cancer
ovarian cancer
method
IVW (Mod.2nd)
MR-RAPS
PRESSO
Weighted median
Weighted mode
MR Egger

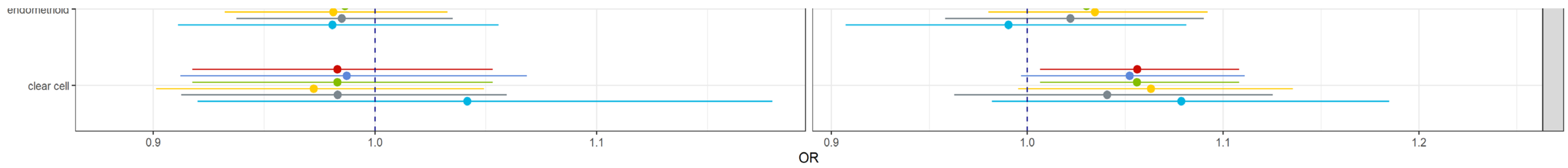

Supplementary Figure 2 Mendelian randomization sensitivity analyses investigating the impact of ankylosing spondylitis on female-specific cancer-risk. Causal estimates represent odds ratios and 95 % confidence intervals.

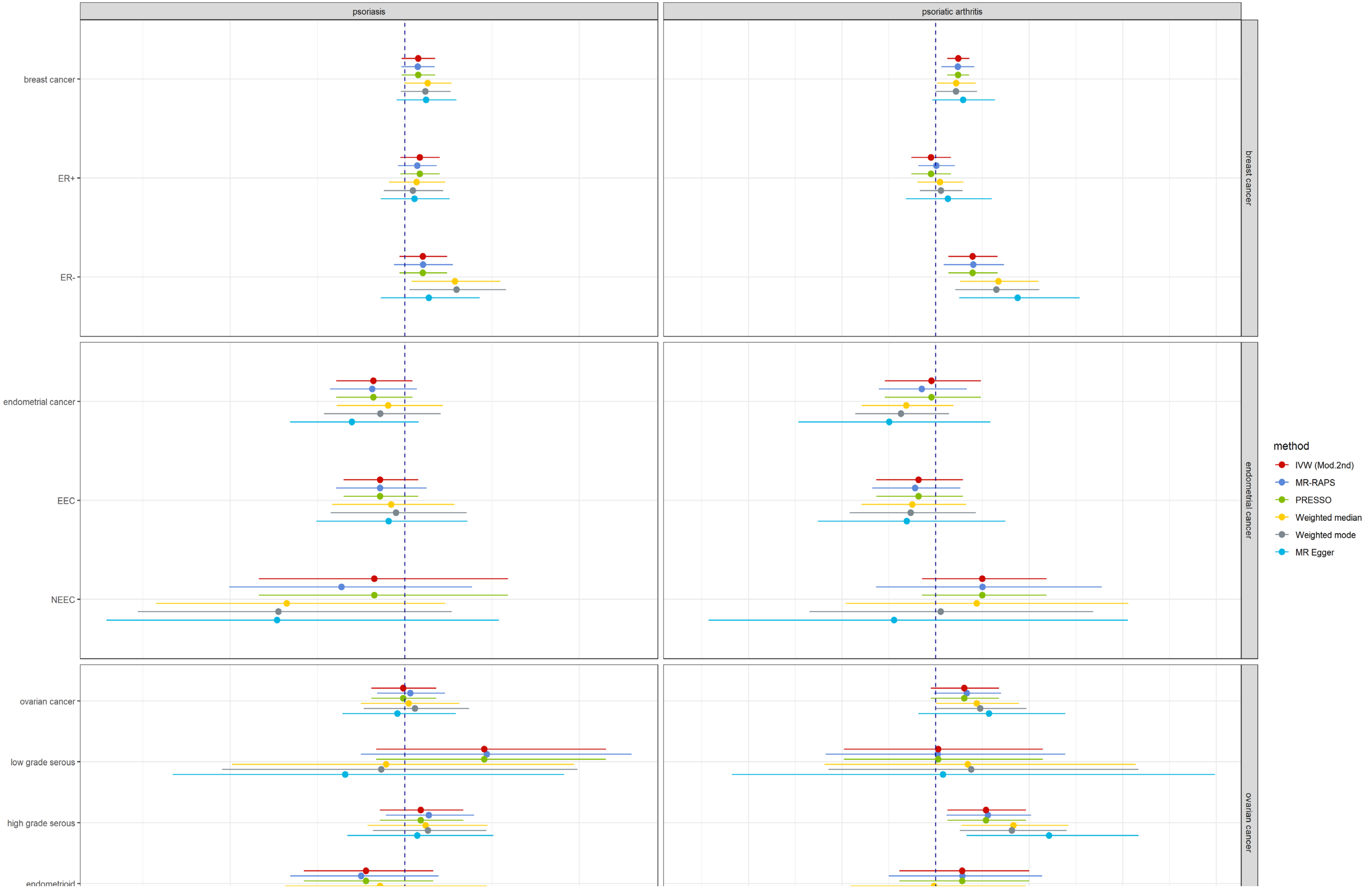

psoriasis
psoriatic arthritis
breast cancer
ER+
ER-
endometrial cancer
EEC
NEEC
ovarian cancer
low grade serous
high grade serous
endometrioid
method
IVW (Mod.2nd)
MR-RAPS
PRESSO
Weighted median
Weighted mode
MR Egger

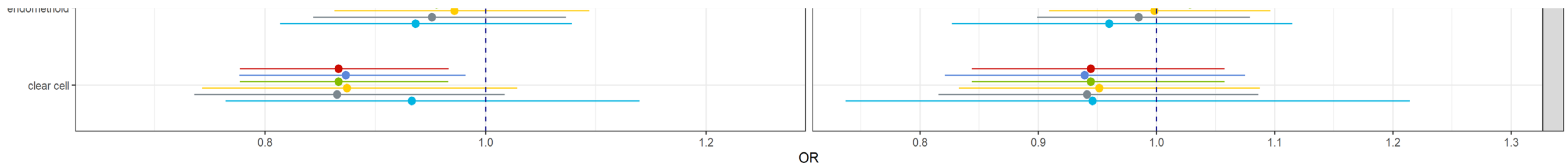

Supplementary Figure 3 Mendelian randomization sensitivity analyses investigating the impact of psoriasis and psoriatic arthritis on female-specific cancer-risk. Causal estimates represent odds ratios and 95 % confidence intervals.

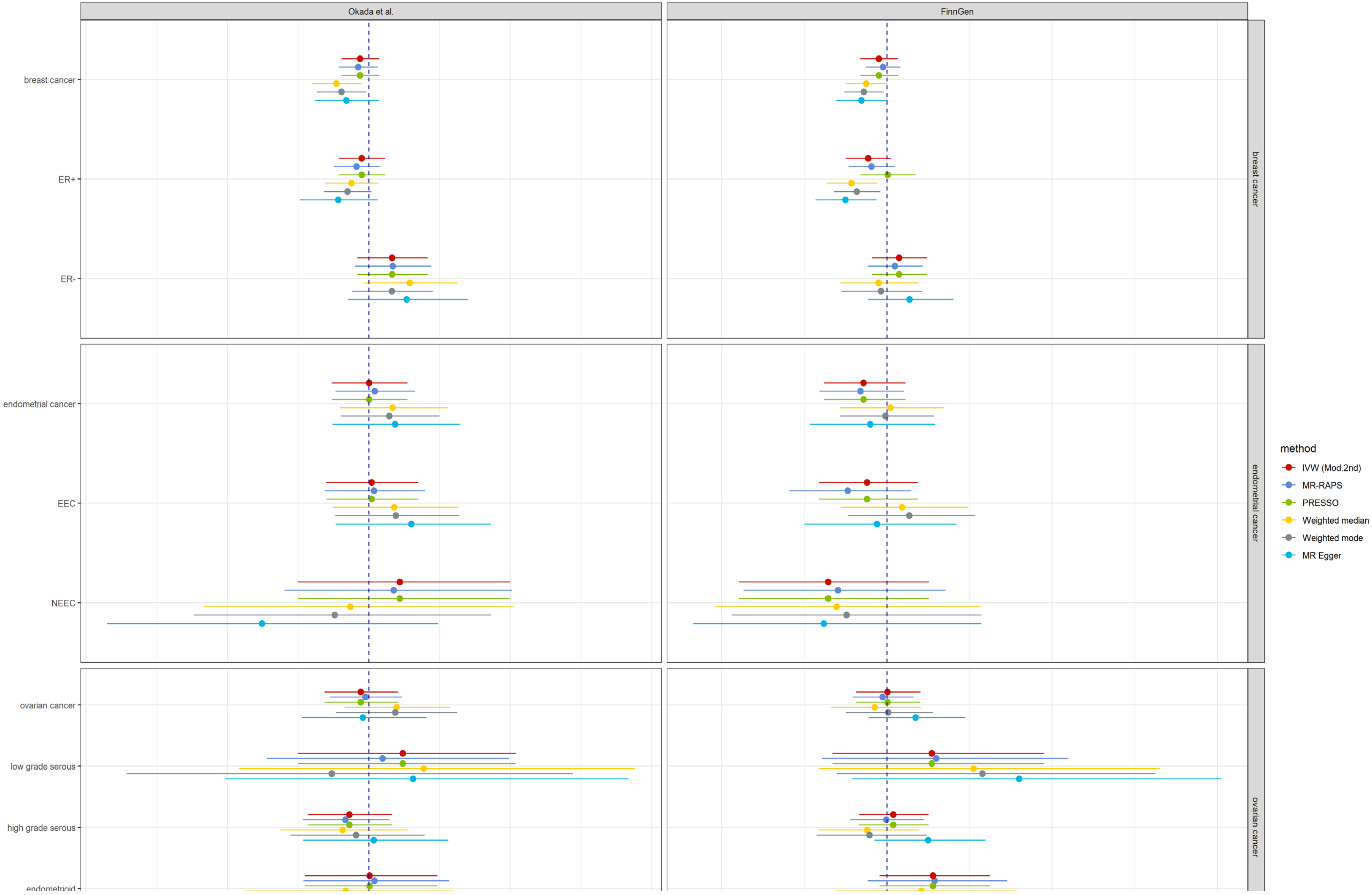

Okada et al.
FinnGen
breast cancer
ER+
ER-
endometrial cancer
EEC
NEEC
ovarian cancer
low grade serous
high grade serous
endometrioid
breast cancer
endometrial cancer
ovarian cancer
method
IVW (Mod.2nd)
MR-RAPS
PRESSO
Weighted median
Weighted mode
MR Egger

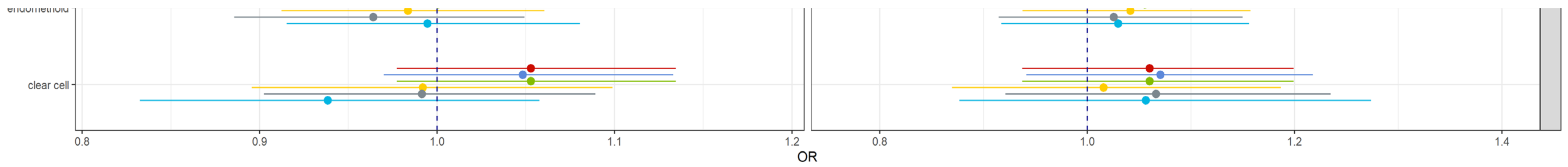

Supplementary Figure 4 Mendelian randomization sensitivity analyses investigating the impact of rheumatoid arthritis on female-specific cancer-risk. Causal estimates represent odds ratios and 95 % confidence intervals.
